# Single-session esophagogastroduodenoscopy and endoscopic ultrasound using a forward-viewing radial scan ultrasonic endoscope

**DOI:** 10.1186/s12876-019-1141-7

**Published:** 2019-12-18

**Authors:** Daisuke Uchida, Hironari Kato, Kazuyuki Matsumoto, Yuki Ishihara, Akihiro Matsumi, Yosuke Saragai, Saimon Takada, Shuntaro Yabe, Shinichiro Muro, Takeshi Tomoda, Shigeru Horiguchi, Hiroyuki Okada

**Affiliations:** 1 Department of Gastroenterology, Okayama University Hospital, 2-5-1 Shikata-cho, Kita-ku, Okayama, 700-8558 Japan; 2 Center for Innovative Clinical Medicine, Okayama University Hospital, 2-5-1 Shikata-cho, Kita-ku, Okayama, 700-8558 Japan

**Keywords:** Endoscopic ultrasound, Diagnostic screening program, Pancreatic diseases, Biliary tract diseases

## Abstract

**Background:**

Endoscopic ultrasound is useful for obtaining high-resolution images of pancreaticobiliary diseases, but is not readily available for physical checkups. In this study, we evaluated the safety and efficacy of single-session esophagogastroduodenoscopy and endoscopic ultrasound in the detection of upper-gastrointestinal and pancreaticobiliary diseases using a forward-viewing radial scan ultrasonic endoscope.

**Methods:**

A total of 148 patients who were scheduled for upper-gastrointestinal screening using an endoscope were prospectively included. All patients were examined by EUS in combination with EGD using a forward-viewing radial scan ultrasonic endoscope. The primary endpoint was the safety of the procedures. The secondary endpoints were the prevalence of diseases, the basal imaging capability of EUS, the procedure time, total dose of propofol, and the correlation between background factors and the prevalence of pancreatic disease. The imaging capability at each region was scored as 0 (invisible) to 2 (sufficient visualization to evaluate the organs).

**Results:**

Intraoperative hypotension occurred as an adverse event of intravenous anesthesia in one patient. There were 82 pancreaticobiliary findings and 165 upper-gastrointestinal findings (malignancy not included). Follicular lymphoma of the intra-abdominal lymph nodes was detected in one patient. The mean imaging scores of each section were 1.95 (pancreatic head and papilla), 2.0 (pancreatic body), 1.99 (pancreatic tail), and 1.89 (common bile duct and gallbladder). Age, history of diabetes mellitus, and smoking history were significantly associated with the prevalence of pancreatic diseases.

**Conclusion:**

The simultaneous performance of EGD and EUS using a new ultrasonic endoscope is tolerable and safe for upper-gastrointestinal and pancreaticobiliary screening.

## Background

It is often difficult to diagnose pancreaticobiliary diseases, including malignant tumors such as pancreatic cancer (PC) and biliary cancer (BC), which exhibit a poor prognosis [1]. In particular, the early diagnosis of PC is an urgent issue because the number of patients with this condition increases each year. Recently, the diagnosis and treatment of PC have advanced, but less than 10% of patients are expected to survive for 5 years after the diagnosis [2]. Patients with early-stage PC often have no subjective symptoms, and most are diagnosed incidentally. Thus, a screening test is required for patients with risk factors for PC; however, the optimal approach is not known.

The detection of premalignant lesions is one solution for improving the survival of PC patients. Transabdominal ultrasound is a non-invasive technique; however, screening of the pancreas is sometimes difficult [3]. Endoscopic ultrasound (EUS) is a novel technique for screening of the pancreas, and is suitable for screening high-risk individuals, and various studies have reported the diagnostic performance of EUS [4–7]. However, it is not a routine test, because it requires dedicated endoscopes, (e.g., endoscopes for radial and linear EUS) [8].

Recently, a new endoscopic ultrasonic processor (SU-1; FUJIFILM, Tokyo, Japan) and radial scan ultrasonic endoscope (EG-580UR; FUJIFILM, Tokyo, Japan) equipped with a direct forward view, which enables the simultaneous performance of esophagogastroduodenoscopy (EGD) and EUS. This endoscope enables the performance pancreaticobiliary screening for patients who undergo EGD. We conducted a prospective intervention study evaluating the efficacy of the new ultrasonic endoscope in upper-gastrointestinal and pancreaticobiliary screening.

## Methods

### Study design and sites

This was a prospective, interventional study conducted at Okayama University Hospital from May 2017 to December 2018. This study was registered in the UMIN protocol registration system (identification number: UMIN000026627).

### Participants

After receiving approval for the study from the institutional review board, we recruited consecutive patients who visited Okayama University Hospital to undergo esophagogastroduodenoscopy (EGD).

The inclusion criteria were as follows:

1. Scheduled for screening EGD.
2. Over fifty years of age (because the prevalence of PC increases with age, especially at ≥50 years of age [9]).
3. The provision of voluntary written consent for participation in this study.

The exclusion criteria were as follows:

1. ECOG performance status 3 or 4.
2. Suspected gastrointestinal bleeding.
3. A history of upper gastrointestinal surgery.
4. Allergy to propofol.
5. Received abdominal computed tomography (CT) or magnetic resonance imaging (MRI) within the past six months.
6. Judged by the attending physician to be ineligible for inclusion in this study).

### Instruments

**Fig. 1:**
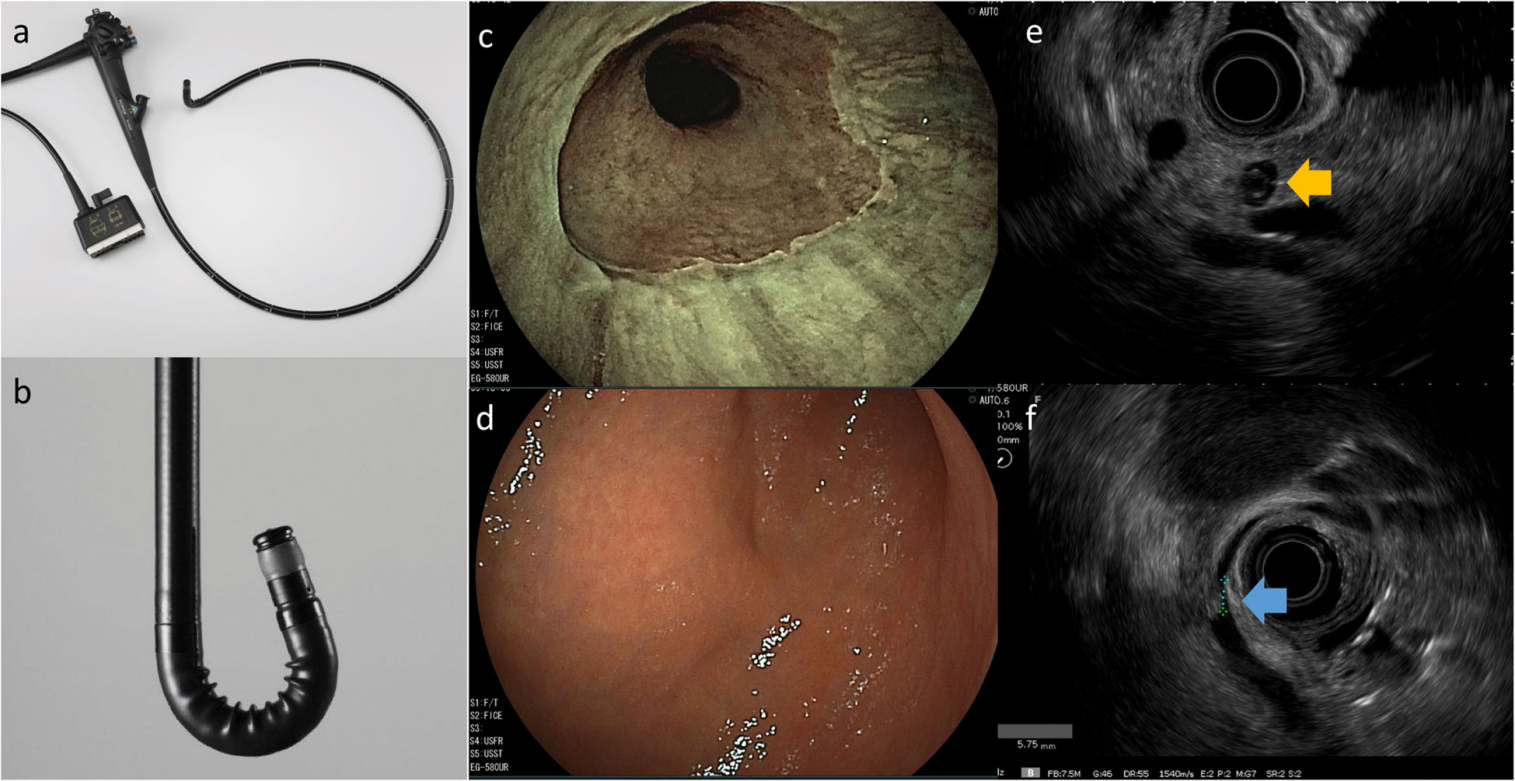
**a** Shape of the EG-580UR. **b**: The diameter of the distal end of the 580UR is 11.4 mm, This device has a the unique ability of bending to 190°, and has a 2.8 mm working channel. **c**: A FICE image of esophagogastric junction obtained using the EG-580UR. **d**: A white light image of the gastric body obtained using the EG-580UR. **e**: An EUS image of the pancreatic body obtained using the EG-580UR. Orange arrow indicates a pancreatic cyst. **f**: An EUS image of the common bile duct obtained using the EG-580UR. Blue arrow indicates a bile duct stone

Forward-viewing radial scan ultrasonic endoscope (EG-580UR; FUJIFILM, Tokyo, Japan) instruments and an endoscopic ultrasonic processor (SU-1; FUJIFILM, Tokyo, Japan) were used. The EG-580UR has an outer diameter of 11.4 mm, a unique capability of bending to 190°, a 2.8 mm working channel, and the super-CCD image quality (Fig. 1). It also has a flexible imaging color enhancement (FICE) mode that can maximize color differences, such as vascular and mucosal patterns, without the need for tissue staining [10]. EUS images were delineated in standard B-mode, and the frequency was set at 7.5 MHz.

### Experimental methods

All procedures were carried out by six experienced endosonographers (DU, HK, KM, YS, ST, and SM), who met all of the following criteria: ≥8 years of endoscopy experience, and ≥ 200 EUS examinations performed > 3 years. Procedures were performed under conscious sedation with propofol (0.8 mg/kg induction dose and a 3–5 mg/kg/h maintenance dose of 1% propofol using an infusion pump, 0.5 mg/kg additional proper boluses). At first, EGD was performed. After EGD screening, EUS screening was performed to evaluate the pancreas, biliary tract, and gallbladder. If abnormal findings were detected, appropriate examinations or treatments were provided.

### Definitions and outcome measures

The primary endpoint was the safety of the procedures. The secondary endpoints were the prevalence of pancreaticobiliary diseases that were detected by EUS, the prevalence of upper-gastrointestinal diseases, the basal imaging capability in the evaluation of the pancreaticobiliary region, the procedure time, the total dose of propofol, and the correlation between the prevalence of pancreatic diseases and patient background. To evaluate the basal imaging capability, the pancreaticobiliary region was divided into four areas; pancreas head (including papilla), pancreas body, pancreas tail, and biliary tract including gallbladder. They were assessed and assigned one of three scores: 0–2 points. “0” was assigned when imaging was completely invisible for all areas; “1” was assigned when the image was partially delineated; and “2” was assigned when the visualization of the target image was sufficient for the evaluation of the organs (Table 3).

Partially visible (score “1”) was defined as any visible delineation that did not meet the criteria for score “2”. For example, if the junction of the superior mesenteric vein (SMV), portal vein (PV), and splenic vein (SPV) was unclear despite the pancreatic body parenchyma being visible, the imaging score of the pancreatic body was “1”. As another example, if the region adjacent to the splenic hilum and left kidney was unclear, despite the pancreatic tail parenchyma being visible, the imaging score of the pancreatic tail was “1”.

### Statistical analysis

All statistical analyses were performed using the JMP Pro 13.0 software program. Categorical data were evaluated with the chi-squared test. *P* values of < 0.05 were considered to indicate statistical significance. A multivariate logistic regression analysis was performed to analyze associations between patient characteristics (items that were associated with prevalence in a univariate analysis; *P* < 0.05) and the prevalence of pancreatic diseases.

## Results

### Patient characteristics

6033 patients underwent EGD screening within the time frame, and 5885 patients were excluded because they were < 50 years of age (*n* = 722), refused to participate (*n* = 836), had a history of upper-gastrointestinal surgery (*n* = 331), or had a history of abdominal CT or MRI within the past six months (*n* = 3996). Finally, a total of 148 patients were included. The patient characteristics are summarized in Table 1. One hundred six of the participants were female (71.6%). Eleven participants (7.4%) had one first- or second-degree blood relative with PC. Twenty-eight (23.3%) participants had a history of diabetes mellitus. The most common reason for EGD screening was follow-up of chronic gastritis due to *Helicobacter pylori*. There were six post-cholecystectomy patients. Forty-four patients had undergone transabdominal ultrasonography (AUS) in the past year.

**Table 1:** Patient characteristics (*n* = 148)

Characteristics	Value
Gender	
Female	106 (71.6%)
Age, median (range)	69 (51–76)
Family history of PC	11 (7.4%)
History of diabetes mellitus	28 (23.3%)
Smoking status	
Never	118
Former	21
Daily	9
Alcohol status	
Daily drinker	44 (29.7%)
Daily alcohol consumption (g), median of drinker	7
Reason for EGD screening	
Follow-up of chronic gastritis due to *Helicobacter pylori*	94
Follow-up of gastric polyp	16
Check for varices for patients with hepatitis	23
Check for reflux esophagitis	9
Abdominal discomfort	6
Post-cholecystectomy	6

*PC* pancreatic cancer; *EGD* esophagogastroduodenoscopy

### Adverse events

One patient had hypotension (systolic blood pressure < 70 mmHg) during the endoscopic procedure. This might have been due to oversedation with propofol. Hypotension was rapidly reversed following the administration of ephedrine (4 mg). She recovered from anesthesia without any further adverse events.

### Prevalence of pancreaticobiliary disease

The prevalence of disease is shown in Table 2. There were thirty-two patients with pancreatic cyst. The median cyst size was 5 mm. The largest was a 20-mm multilocular cyst, which was diagnosed as a branch duct intraductal papillary mucinous neoplasm (IPMN) without high-risk stigmata or worrisome features. All patients with pancreatic cysts were scheduled for follow-up MRI at six months. Twenty-five patients were diagnosed with early chronic pancreatitis, which had 3 to 4 minor features of chronic pancreatitis, according to the Rosemont classification [11]. No patients had symptoms of chronic pancreatitis. Fourteen patients had gallbladder polyp (suspected cholesterol polyp). Nine patients had adenomyomatosis and thirteen had asymptomatic gallbladder stones. These gallbladder lesions were scheduled for follow-up AUS at six months. One patient had asymptomatic common bile duct stone, and was treated with endoscopic removal of the common bile duct stone by endoscopic sphincterotomy. Other patients were followed up with AUS, MRI or CT at one year at the patient’s request.

**Table 2:** Prevalence of diseases

Pancreaticobiliary diseases	
Pancreatic cysts	32 (21.6%)
Size of cyst, median, range (mm)	5 (2–20)
Early chronic pancreatitis*	25 (16.9%)
Gallbladder polyp	14 (9.5%)
Size of polyps, median range (mm)	4 (1–5)
Adenomyomatosis	9 (6.1%)
Gallbladder stone	13 (8.8%)
Common bile duct stone	1 (0.7%)
Upper-gastrointestinal diseases	
Barrett’s esophagus	9 (6%)
Reflux esophagitis	41 (27.7%)
Candida esophagitis	1 (0.7%)
Esophageal papilloma	1 (0.7%)
Esophageal submucosal tumor	2 (1.4%)
Chronic gastritis	69 (46.6%)
Hyperplastic polyp	9 (6%)
Fundic gland polyp	14 (9.5%)
Gastric ulcer scar	1 (0.7%)
Gastric submucosal tumor	8 (5.4%)
Duodenal ulcer scar	8 (5.4%)
Duodenal submucosal tumor	2 (1.4%)
Others detected by EUS	
Follicular lymphoma of intra-abdominal lymph nodes	1 (0.7%)

*EUS* endoscopic ultrasound

Forty-four patients had undergone AUS in the past year. Sixteen of these patients were diagnosed with pancreatic disease (pancreatic cyst, *n* = 8; early chronic pancreatitis, n = 8) which was not detected by AUS in the past year. Twelve of these patients were diagnosed gallbladder disease (adenomyomatosis, *n* = 7; gallbladder stones, *n* = 5), that had already been diagnosed by AUS. To date, 88 (59.5%) have already undergone follow-up CT or MRI examinations. None of the patients showed other pancreaticobiliary findings.

### Prevalence of upper-gastrointestinal disease

As shown in Table 2, various findings were detected by EGD screening. No patients had malignant features. In 56 patients (37.8%) cases, it was difficult to delineate the lesser curvature of the gastric angle when pulling back the scope because of the long distal end of the scope. In these cases, the gastric angle could be evaluated in the short scope position. All patients were scheduled for follow-up EGD at one year. To date, 103 patients (69.6%) have already undergone follow-up EGD using a conventional EGD scope. Fortunately, no patients had other findings on follow-up EGD.

### Other diseases detected by EUS

There was one patient with follicular lymphoma of the intra-abdominal lymph nodes. A swollen abdominal lymph node (> 25 mm) was pointed out, and EUS-guided fine needle aspiration (EUS-FNA) was performed. EUS-FNA revealed follicular lymphoma. She was followed closely by hematologists.

### Basal imaging capability of EUS

The mean imaging scores of each section were 1.95 (pancreatic head and papilla), 2.0 (pancreatic body), 1.99 (pancreatic tail), and 1.89 (common bile duct and gallbladder) (Table 3). The biliary imaging score of post-cholecystectomy patients was without gallbladder imaging. There were no patients with score “0” in any section.

**Table 3:** Basal imaging capability of EUS

	Score definition			Mean score
	2	1	0	
Pancreatic head and papilla	Duodenal papilla (the region of confluence of the pancreatic duct and bile ducts on the duodenal muscularis), and the pancreas head (the region surrounded by the SMA and the scope) are clearly visible	Partially visible	Completely invisible	1.95
Pancreatic body	The pancreatic body (the region from the proximal parenchyma of the junction of SMV, PV and SPV up to pancreatic tail) is clearly visible	Partially visible	Completely invisible	2
Pancreatic tail	Pancreatic tail (the region adjacent to the splenic hilum and left kidney) is clearly visible	Partially visible	Completely invisible	1.99
Common bile duct and gallbladder	Common bile duct (from confluence of hepatic duct to pancreatic bile duct including junction of cystic duct), and gallbladder (from neck to fundus) are serially visible	Partially visible	Completely invisible	1.89

*EUS* endoscopic ultrasound; *SMA* superior mesenteric artery; *SMV* superior mesenteric vein; *PV* portal vein; *SPV* splenic vein

### Procedure times and total dose of propofol

The median procedure time was 22 min (EGD, 10 min; EUS, 11 min). The median total dose of propofol was 145 mg. A breakdown of these results is shown in Table 4.

**Table 4:** Procedure time, and total dose of propofol

Procedure time (min); median and range			Total dose of propofol (mg); median and range
EGD	EUS	total	145 (30–350)
10 (5–25)	11 (10–29)	22 (15–45)	

*EGD* esophagogastroduodenoscopy; *EUS* endoscopic ultrasound

Correlation between the prevalence of pancreatic disease and patient background factors.

Age and a history of diabetes mellitus were extracted as factors significantly associated with the prevalence of pancreatic cysts in a univariate analysis. In the multivariate logistic regression analysis, age was the only significant factor. Sex, history of diabetes mellitus, smoking history, and daily alcohol were extracted as factors significantly associated with the prevalence of early chronic pancreatitis. In the multivariate logistic regression analysis, history of diabetes mellitus and smoking history were significant factors. Detailed data are shown in Table 5.

**Table 5:** The correlation between the prevalence of pancreatic diseases and patient background

	Pancreatic cyst (*n* = 32)					Early chronic pancreatitis (*n* = 25)				
	Number	Univariate		Multivariate		Number	Univariate		Multivariate	
		Odds ratio (95%CI)	*P* value	Odds ratio (95%CI)	*P* value		Odds ratio (95%CI)	P value	Odds ratio (95%CI)	*P* value
Age										
≥ 70 (*n* = 68)	22 (32.4%)	3.35 (1.45–7.71)	0.0035	3.18 (1.36–7.38)	0.0073	12 (17.7%)	1.10 (0.47–2.61)	0.8212		
< 70 (*n* = 80)	10 (12.5%)					13 (16.3%)				
Sex										
Male (*n* = 42)	9 (21.4%)	0.98 (0.41–2.35)	0.9714			14 (33.3%)	4.32 (1.76–10.6)	0.0008	2.08 (0.65–6.66)	0.2147
Female (*n* = 106)	23 (21.7%)					11 (10.4%)				
Family history of PC										
Yes (*n* = 11)	4 (36.4%)	2.22 (0.61–8.14)	0.217			2 (18.2%)	1.10 (0.22–5.44)	0.9055		
No (*n* = 137)	28 (20.4%)					23 (16.8%)				
History of diabetes mellitus										
Yes (*n* = 28)	10 (35.7%)	2.47 (1.01–6.09)	0.0442	2.23 (0.88–5.65)	0.0921	10 (35.7%)	3.89 (1.51–9.99)	0.0032	3.78 (1.33–10.8)	0.0128
No (*n* = 120)	22 (18.3%)					15 (12.5%)				
Smoking history										
Yes (*n* = 30)	6 (20%)	0.88 (0.33–2.39)	0.8091			12 (40%)	5.38 (2.12–13.7)	0.0002	3.54 (1.04–12)	0.043
No (*n* = 118)	26 (22%)					13 (11%)				
Alcohol status										
Daily (*n* = 44)	11 (25%)	1.32 (0.57–3.03)	0.5161			13 (29.6%)	3.22 (1.33–7.78)	0.0075	1.06 (0.34–3.3)	0.9223
Never or occasional (*n* = 104)	21 (20.2%)					12 (11.5%)				

*PC* pancreatic cancer

## Discussion

Small pancreaticobiliary tumors, such as PC and BC, are difficult to diagnose because they have minimal symptoms in the early stages. However, advanced pancreaticobiliary cancers exhibit a poor prognosis. The 5-year survival rate of PC patients is approximately 8%, and PC is the fourth leading cause of cancer death worldwide [12, 13]. The early diagnosis of PC is one solution to improving the prognosis of PC, and various approaches have been developed such as genetic screening of high-risk patients [14, 15].

EUS is considered to be a sensitive device for the diagnosis of pancreaticobiliary diseases [16–18]. In particular, curved linear array EUS can obtain tissue by EUS-guided fine needle aspiration (EUS-FNA), and has become the scope that is predominantly used for EUS examination. Meanwhile, radial scan EUS is used for screening examinations, and can obtain transverse images of the pancreas, which provides objective evaluations. However, these scopes are dedicated EUS devices, and are unsuitable for medical check-ups.

On the other hand, EGD using a forward-viewing endoscope and transabdominal ultrasound are commonly used in medical check-ups in Japan [19]. A forward-viewing radial scan ultrasonic endoscope and ultrasound processor (SU-1 and EG-580UR, respectively; FUJIFILM, Tokyo, Japan) enable single-session screening of the upper gastrointestinal tract and pancreaticobiliary organ. EUS provides high-resolution images, and is anticipated to be highly useful in the early diagnosis of pancreaticobiliary tumors [16]. We conducted this study to evaluate the efficacy of this device for the early diagnosis of pancreaticobiliary diseases, especially pancreatic malignancies.

In the present study, we recruited 148 patients scheduled for EGD screening (Table 1). We defined the minimum age as 50 years, because the prevalence of PC increases with age, especially in patients of ≥50 years of age [9]. As a result, a comparatively large number of findings are detected by procedures (Table 2). EGD screening revealed various findings, including reflux esophagitis, chronic gastritis, and gastrointestinal benign tumors, but not malignancies. The image quality of the CCD of the EG-580UR device is not equivalent to that of the latest endoscopes. In fact, to delineate the lesser curvature of the gastric angle when pulling back the scope was difficult in 56 patients (37.8%). This constructional disadvantage should be improved.

Various findings were detected by EUS screening. As shown in Table 2, pancreatic cysts, which were detected in 32 patients, were the most common finding.

Incidental pancreatic cysts are reported to be associated with increased mortality, and follow-up is recommended for patients in whom they are identified [20, 21]. IPMNs are pancreatic cysts that are associated a risk of malignancy; however, pancreatic cysts other than IPMNs may cause pancreatic ductal carcinoma [22]. Laffan et al. reported that the prevalence of unsuspected pancreatic cysts detected by MDCT in an outpatient population was 2.6%, which was correlated with increasing age and Asian race [23]. There are also some reports on the prevalence of pancreatic cysts on MRI imaging. De Jong reported that the prevalence was 2.4%, while Lee et al. and Zhang et al. reported that the prevalence was 13.5 and 19.6%, respectively [24–26].

EUS is undoubtedly a superior tool for the diagnosis of pancreatic diseases, including pancreatic cysts. Kamata et al. reported that the EUS was superior to other imaging modalities (e.g., CT or MRI) for the early detection of PC in patients with IPMN [27]. Pausawasdi et al. reported that EUS offered some benefits in the evaluation of pancreatic cyst [28]. They referred to the possibility of the molecular and biomarker analysis of cyst fluid obtained by EUS-FNA. Barresi et al. reported the efficacy of EUS-through-the-needle biopsy in pancreatic cystic lesions; however, this procedure is still in the investigational stage, because it is associated with a risk of needle tract seeding [29]. Additionally, contrast-enhanced EUS is reported to be an effective tool for the diagnosis of pancreatic cysts [30, 31]. We consider that EUS with high-resolution imaging can be a tool for identifying and qualitatively diagnosing pancreaticobiliary diseases.

Our result showed that the prevalence of pancreatic cysts was 21.6%, which was clearly higher in comparison to previous reports. This contradictory finding have been due to the fact that our cohort consisted of patients of ≥50 years of age. Actually, an autopsy study revealed that 24.3% of patients (most patients were ≥ 65 years of age) had pancreatic cysts [32]. Fortunately, there were no malignant findings, such as high-risk stigmata or IPMNs with worrisome features, and the patients with cysts of > 5 mm in size were scheduled for follow-up MRI at six months. In our data, age and a history of diabetes mellitus were significantly associated with the prevalence of pancreatic cysts (Table 4).

Chronic pancreatitis is a risk factor for PC [33]. Recently, the early diagnosis of chronic pancreatitis with EUS has gained attention; however, the extent to which early chronic pancreatitis is associated with pancreatic carcinogenesis is still unclear [11, 34]. In our study, the prevalence of early chronic pancreatitis was 16.9%; this was related to a history of diabetes mellitus and smoking history (Tables 2 and 4). This result is in line with previous reports, and serves as a useful reference for identifying high-risk patients [24, 34–36]. A family history of PC is a known risk factor for PC [37, 38]. There was no significant association between a family history of PC and the prevalence of pancreatic disease in this study. There may be some reasons for this controversial result. Canto et al. reported that individuals with three or more blood relatives with PC, including at least one first-degree relative, should be considered for screening [38]. In our study, there were 11 patients with only one affected first or second-degree blood relative. Additionally, the relatively small number of patients may have affected the result.

One patient had enlarged intra-abdominal lymph nodes, and was diagnosed with follicular lymphoma by EUS-FNA and 18-fluoro-2-deoxy-D-glucose positron emission tomography (FDG-PET). She was referred to a hematology specialist, and followed closely without chemotherapy.

The imaging capability of EUS was generally good (Table 3). However, these procedures were performed by experienced endosonographers. The technique of EUS imaging is sometimes difficult, and the diagnostic performance of EUS is operator-dependent. Increasing the number of endosonographers is also necessary to facilitate the early diagnosis of pancreaticobiliary diseases.

The screening method using the EG-580UR device is a little different from the traditional method using an ordinary EUS scope, especially with regard to duodenal manipulation, because this new scope has a slim distal end (11.4 mm) and a small bending radius. At first, this difference perplexed us; however, all operators soon got used to it. Actually, the imaging capability of EUS was generally good, and the procedure time was tolerable (Tables 3 and 4). Recently, curved linear array EUS scope is widely used. Radial scan EUS scope provides understandable organ image with 360°scanning range, however it cannot collect tissue samples. Kaneko et al. reported that there was not a significant difference between the imaging capability of radial scan scope and curved linear array scope, however, both scope have pros and cons [39]. They reported radial scan scope was superior in the delineation of the major duodenal papilla and gallbladder. Front-viewing radial scan scope may be able to expand options for scope choice. Additionally, this scope and single-session EGD and EUS method may widen the training opportunities for novices. Increasing experts of EUS procedure using forward-viewing radial scan EUS scope may be conductive to improve pancreaticobiliary diseases.

The present study was associated with some limitations. The accuracy of the EUS findings was unclear because some participants did not undergo screening with other modalities (e.g., CT or MRI). Most patients were also scheduled for follow-up AUS, MRI or CT at six months; however, some patients who had no pancreaticobiliary findings did not wish to undergo the examination because of the high cost. Fortunately, there were no other pancreaticobiliary findings in 88 patients (59.5%) who—at the time of writing—have already undergone follow-up using AUS, CT or MRI. Additionally, the accuracy of EGD was still unclear. To date, 103 patients (69.6%) have already undergone follow-up EGD using a conventional EGD scope, and no other gastrointestinal findings were identified in follow-up EGD. It was unclear whether EUS was superior to other modalities; however, some patients were diagnosed with pancreatic diseases that could not be detected by post-AUS. This result proved the high sensitivity of EUS in the diagnosis of pancreatic lesions. These subjects should be evaluated in a future analysis with comparative design including large number of participants.

In this study, there was only one adverse event, which might have been caused by oversedation. We were concerned about the risk of increasing the dose of propofol; thus, we excluded patients with an ECOG performance status of 3 or 4. However, most procedures were completed within 30 min as a result. This might have been due to the normal EUS findings in most patients.

## Conclusion

In conclusion, a forward-viewing radial scan ultrasonic endoscope, the EG-580UR, was a novel tool for screening of gastrointestinal and pancreaticobiliary diseases. Further developments in manipulation and image quality are expected, which may help to improve the prognosis of pancreaticobiliary diseases.

## Abbreviations

AUS: Transabdominal ultrasonography
BC: Biliary cancer
CT: Computed tomography
EGD: Esophagogastroduodenoscopy
EUS: Endoscopic ultrasound
EUS-FNA: Eus-guided fine needle aspiration
FDG-PET: 18-fluoro-2-deoxy-D-glucose positron emission tomography
FICE: Flexible imaging color enhancement
IPMN: Intraductal papillary mucinous neoplasm
MRI: Magnetic resonance imaging
PC: Pancreatic cancer
PV: Portal vein
SMV: Superior mesenteric vein
SPV: Splenic vein
